# Enormous Swelling of the Scrotum, Resembling Elephantiasis, with the Operation

**Published:** 1830-04-01

**Authors:** 


### XLIV.

### CASE OF AN ENORMOUS SWELLING OF THE SCROTUM, WITH SYMPTOMS OF ELEPHANTIASIS. *

Jean Baptiste Authier, married, a tall, dark, well-proportioned man, of a strong bilious constitution, 35 years of age, born at Perpignan, in the Eastern Pyrennees. His father died of an acute affection in his 55th year, and was like himself, of an athletic habit. His mother is also strongly constituted, and now in her 63d year, never having been seriously ill. Authier enjoyed good health until 14 years of age, at which time he entered a regiment of light troops, and followed the French army into Portugal. After four years' service he returned home and pursued his trade of a baker, at which period he caught a gonorrhœa, which yielded to mucilaginous drinks and mercury. At 25 years of age he was admitted into the Gendarmerie à Cheval, two years after which there appeared a slight ulceration on the prepuce; this was immediately treated by caustics, which caused great pain and intense inflammation. Authier obtained permission to return to his family for three months, where he underwent a mercurial course by frictions. At the expiration of the three months he wished to rejoin his corps although the ulceration was not quite healed; the fatigue of riding soon caused a return of the pain and inflam-

* From Professor Delpech's Clinique Chirurgicale de Montpellier.

mation; the swelling increased and in the course of two months extended over the whole prepuce. But it became indolent, and the skin infiltrated, hard, tuberculated, and furrowed with deep wrinkles. All the skin of the scrotum, particularly of the lower part, lost its consistency, and became gradually brown, hardened, thickened, tuberculated, and deeply wrinkled. The subjacent cellular tissue was also considerably swelled, feeling first doughy, afterwards consistent, hard and heavy; its weight drew the scrotum down, distending the cutaneous sheath of the penis; and this part of the integuments became inverted in proportion, the penis disappearing and becoming as if sheathed in the tumour. The swelling of the cellular tissue made it difficult to distinguish the testicles, which became daily more deeply seated. In this state Authier could no longer endure riding; he left the Gendarmerie and returned to his family; and during the following year the tumour rapidly increased, and its lower part became very irregular. During the last three years, the tumour, which was no longer of a doubtful nature, increased much less rapidly than previously. At this time he went into the hospital at Perpignan, where some mercurial preparations were made use of during a short time. He was shortly after sent to Montpellier, where he was admitted into the Hospital St. Eloi, at the commencement of July, 1820. The disease had existed seven years, and his present condition was as follows:—It was evident that the skin of the penis and scrotum, as well as the cellular tissue of the latter part, were affected with elephantiasis; but there was no symptom of the same affection in any other part of the body. The tumour was pyriform, flattened transversely, divided into three lobes at its lower anterior part, and extended below the calf of the leg with a large eminence projecting backwards and held to the perinæum and hypogastric regions by a neck or pedicle, which occupied the whole space between the pubic region, the two groins, and the anus. This neck was 18 inches in circumference at its smallest part; it was larger in front where it covered the two external abdominal rings and the spermatic cord, which could not be distinguished externally; it was less extended posteriorly, where it seemed to be compressed by the buttocks to the size of the perinæum. At the lateral and posterior parts of this neck the skin appeared healthy, supple, and was thin, from the distention it had undergone. On the fore part as far as the pubes, it was much thicker and of such a consistency that, when the patient was laid horizontally, folds could be formed of the skin, in the substance of which the infiltration of the subjacent cellular tissue could be dispersed by pressure of the fingers. No part of the genital organs could be distinguished in this neck. The skin was hard, knotty, and adherent at the middle of this mass, not changing at all from pressure: the patient declared that he felt as if his testicles were squeezed when the sides of the tumour were pressed together, at about the distance of one foot from the external abdominal rings. The most dependent part was of a reddish brown colour, divided into three principal lobes, which were covered with knobs formed of the skin, which were more numerous here than elsewhere. Under the anterior portion was a great transverse groove, at the bottom of which was situated a deep sinus leading upwards and slightly to the left; this was the prepuce and urethra, and there was nothing above these like a penis, but the patient assured us that he had occasionally erections and emissions. The entire mass when weighed at Perpignan was estimated at above 60lbs. Walking or standing was very painful, the nutritive functions were not at all disturbed, there were no colicky pains, and the volume of the tumour did not vary with that of the abdomen; the breathing was free without cough or expectoration; the general cutaneous surface, excepting the parts of generation, was not at all changed, but supple, smooth, and perspirable, and of the same temperature as the rest of the body. Yet it was very white, the face having a leaden tinge, which the patient declared to be his natural appearance. After maturely reflecting on the chances of the patient's being benefited, the removal of the diseased mass was decided on.

The operation was performed in the following manner. The patient was placed horizontally, exposed to a good light, with the nates on the edge of the bed, and the legs and thighs bent and in a state of abduction. The tumour was held horizontally at its middle circumference, by means of a large cloth, whilst two assistants standing upon the bed, one on each side of the patient, were desired to sustain the tumour and vary its position by means of the girth. We were placed in front of the patient, between his legs, and traced with ink the intended direction of the incisions; each of the two principal ones was to begin near the external abdominal ring, describe in descending a large curve, the sinus of which was turned upwards, pass obliquely over one of the sides of the pedicle of the tumour, and terminate before the anus, at which spot the two incisions were to be united at a tolerably acute angle. The direction of three other incisions was also marked: the two first were curved with the hollow of the curve directed outwards, and commenced on either side under the anterior fifth of the curved lateral incision, and terminated four inches lower down, at a point where a vertical line would have been a tangent to this new curve. The third and last section was traced by a horizontal line which united the inferior extremity of the two last curved incisions. These five incisions were to form three cutaneous flaps. The integuments were divided by means of a convex bistoury, following precisely the lines traced. The two lateral flaps were first dissected as far back as the pubic arch, the fascia lata being laid bare at the base of each of the flaps, because all the cellular tissue the perinæum, and a portion of that at the root of the thigh had been drawn down towards the tumour, and affected to a certain extent in the same manner as the scrotum; the third flap was also dissected down to its base, as far as the pubic arch and the ossa pubis. In the formation of these three flaps we took care to dissect back the skin and cellular tissue that were loose and not infiltrated, which was not practicable with respect to the anterior flap, the dermoid tissue itself being infiltrated with serum. Several ligatures were applied at the base of each flap. Under the fore part of the right lateral flap, and opposite the corresponding abdominal ring, we made an incision into the cellular tissue, which we continued downwards in the direction of the axis of the ring. At the depth of two inches we met with the external pudic vessels, which we divided and tied at the groin. On the opposite side we did the same, and at a still greater depth we found the spermatic cord and vessels; the cremaster appeared thicker, redder, and more distended than usual. We were now at a depth of about four inches in the substance of the tumour, following a diagonal line extending from the left groin towards the right nates; there was a remarkable difference between the dense, lardaceous, partly fibrous tissue of which it was composed, and the loose transparent permeable cellular tissue which immediately enveloped the cord. The spermatic cord was followed by the left finger, which served to conduct the incisions; the testicle was found a little larger than it naturally is, but rather soft, without any effusion into the tunica vaginalis, and more firmly fixed at its posterior extremity to the bottom of the root of cavity which contained it, than the cord at any other part. The patient complained of intolerable pain on pressing the testicle firmly; the left testicle and spermatic cord were detached up to the ring and laid on the abdomen. The right testicle was contained in a larger cavity than the left, easily detached, and laid on the abdomen. The left fore-finger was carried into the sinus at the bottom of the tumour by which the urine passed, and which answered to the orifice of the prepuce; it served to guide the incisions which we made from below upwards for the purpose of dividing this sinus, and the sort of cauliflower lobe which covered its orifice, and thus arrive at the glans, which was found at the height of a foot. The whole of the tumour being thus divided on its fore-part from the root of the glans up to opposite the symphisis pubis, facilitated the dissection, which laid bare the right and left corpus cavernosum. We divided the prepuce round the glans, and dissected up the parts at the posterior part of the penis and urethra, which were only attached to the tumour by loose cellular membrane quite different to the morbid mass; the penis was isolated and placed on the abdomen with the testicles. We now proceeded to the section of the pedicle of the tumour, exposing with ease the different parts of the perinæum; we first discovered the left side of the pubic arch, then the left corpus cavernosum, and successively the canal of the urethra, the bulb, and membranous portion, the erector penis, accelerator urinæ, sphincters of the anus, the right corpus cavernosum, and the right side of the pubic arch, at which part the dissection terminated. On removal of the tumour the artery of the septum of the scrotum, the dorsal arteries of the penis, transverse of perinæum, that of the bulb on either side, and several of the inferior hæmorrhoidal vessels were secured and the ends of the ligatures cut off close to the knots.

The testicles were lodged on the perinæum on either side of the root of the penis. The great length of the spermatic cords necessitated their being placed in zig-zag, in order to confine them in the narrow space between the external abdominal ring and the testicle; it was with difficulty that these parts were kept in situ. The two lateral flaps of integuments were brought together, and maintained so by sutures, as far as the root of the penis, where we omitted the median suture in order to form the sheath of the penis. The anterior flap was rolled round the penis, so that the two sides of the flap which went from the inguinal rings were left free, and the two following were brought together under the whole length of the urethra, and confined at every interval of 8 lines by sutures. The middle portion of this flap, which we may call anterior or inferior, was turned round the extremity of the glans, and represented the free end of the prepuce. The sides of this flap which, between the inguinal ring and the root of the penis on both sides, had been left free, were brought together, each being attached to the anterior fifth of the corresponding lateral flap by sutures, at intervals of 8 lines.

Up to the division of the pedicle of the tumour the operation had lasted 57 minutes. We thought it of importance to relieve the patient's sufferings as soon as possible, and accordingly gave him 60 drops of laudanum in aq. ℥ij. while the sutures were being applied; long narow pledgets of charpie smeared with cerate covered the parts brought into contact and the sutures; charpie was laid over the new scrotum from the anus to the groins; long compresses, crossed behind and separated in front, covered this mass of charpie; and the whole was confined by a double T bandage applied tolerably tight, in order to prevent any excessive swelling, and to keep in contact the parts which now formed the scrotum, the spermatic cords, the testicles, and the new surface of the perinæum; the groin and penis were but slightly compressed. The weight of the tumour an hour after its extirpation was 54lbs, the quantity of serum that oozed during or after the operation was estimated at 6lbs. Immediately after the operation the pulse was almost insensible, with pale face and cold extremities; sloughing of the flap covering the penis, and suppuration in one or two other parts occurred; but on the whole matters went on so well, that the patient had entirely recovered by the end of November, and left the Hospital in February. Very good advice was given by the surgeon, but as soon as he was at home he committed excesses and irregularities of all kinds, was seized with obscure symptoms, and died on the 23d of February. On dissection an abscess was found in the right lobe of the liver, containing about a pint of pus; nothing further of consequence was observed in the abdomen or chest, nor any thing worth transcribing in the genito-urinary apparatus.

Some interesting remarks on elephantiasis of the scrotum are appended by M. Delpech, but after the article on this disease from Dr. Titley's book, which our readers will find in the Periscope of the present number; it is unnecessary for us to dwell any further on the subject.

